# Use of Robotic Surgery for the Management of Orbital Diseases: A Comprehensive Review

**DOI:** 10.3390/medicina61061081

**Published:** 2025-06-12

**Authors:** Riccardo Nocini, Lorenzo Marini, Luca Michelutti, Chiara Zilio, Stefania Troise, Salvatore Sembronio, Giovanni Dell’Aversana Orabona, Massimo Robiony, Alessandro Tel

**Affiliations:** 1 Section of Ear Nose and Throat (ENT), Department of Medical and Surgical Sciences, Dentistry, Gynecology and Pediatrics, University of Verona, 37124 Verona, Italy; riccardo.nocini@gmail.com; 2 Clinic of Maxillofacial Surgery, Head-Neck and Neuroscience Department, University Hospital of Udine, p.le S. Maria della Misericordia 15, 33100 Udine, Italy; marini.lorenzo@spes.uniud.it (L.M.); micheluttiluca.uniud@gmail.com (L.M.); zilio.chiara@spes.uniud.it (C.Z.); massimo.robiony@uniud.it (M.R.); 3 Maxillofacial Surgery Unit, Department of Neurosciences, Reproductive and Odontostomatological Sciences, University of Naples Federico II, 80055 Portici, Italy; stefania.troise@unina.it (S.T.); giovanni.dellaversanaorabona@unina.it (G.D.O.)

**Keywords:** robotic surgery, cranio-maxillo-facial surgery, orbital surgery, precision surgery

## Abstract

*Background and Objectives*: Robotic surgery represents one of the most significant innovations in the field of surgery, offering new opportunities for the treatment of complex pathologies that require greater accuracy and precision. It is a technology that has become widely used in general, urologic, gynecologic, and cardio-thoracic surgery, but has a limited evidence in the head and neck region. This review explores the use of robotic surgery in orbital pathology, focusing on its applications, benefits, and limitations. *Materials and Methods*: A cross-sectional search method was performed in multiple databases to answer the following question: “What are the applications of robotic surgery in the management of orbital pathologies?” Studies were carefully reviewed by two simultaneous researchers, and, in case of disagreement, a third researcher was engaged. Care was taken to identify the surgical hardware (robotic station) used to perform the surgical procedure. *Results*: Out of 491 records, eight studies met the inclusion criteria. These included cadaveric, preclinical, in vitro, and early clinical investigations assessing robotic approaches for fronto-orbital advancement, tumor resection, orbital decompression, and other surgical procedures such as lacrimal gland dissection and biopsy, medial and lateral orbital wall dissections, enucleation, and lid-sparing orbital exenteration. The robotic systems evaluated included the Da Vinci Xi, Da Vinci SP, Medineering Robotic Endoscope Guiding System, and a modular multi-arm concentric tube robot, each with specific advantages and limitations. *Conclusions*: Robotic surgery provides significant advantages for orbital pathologies such as improved precision, visualization, and tissue preservation, with reduced complications and faster recovery, although some limitations still exist. Future advancements, such as smaller instruments and AI integration, promise to improve outcomes, making robotic surgery more effective in treating orbital conditions.

## 1. Introduction

Robotic surgery represents one of the most revolutionary technological innovations in modern medicine. This technology, introduced in the 1980s, has transformed some surgical practices, offering greater precision and control especially for anatomically complex surgeries [1,2,3].

The clinical applications of robotic surgery are broad and include many medical specialties, such as general surgery, gynecology, urology, cardiology, and neurosurgery [4,5,6]. In each of these areas, robotic surgery was demonstrated to offer several significant advantages over traditional surgical techniques, including greater precision of surgical performance, less invasiveness, reduced pain and postoperative complications, better functional and aesthetic outcomes, and faster recovery from surgery [7,8,9,10].

However, despite the many benefits this innovative technology offers, robotic surgery also presents some challenges and limitations. These include the cost of robotic systems and the need for specialized training. In addition, these systems require constant improvements and upgrades [11,12].

Robotic surgery, despite being a technology introduced several years ago, is constantly evolving by integrating artificial intelligence algorithms, haptic feedback, gesture tracking, features which can also provide intraoperative metrics and enable automation of surgical steps with improved motion control. Augmented reality allows surgeons to visualize 3D reproductions of organs and tissues, and updates in 5G connectivity are essential for telesurgery, giving the ability to perform remote surgeries around the world in real time with decreased latency [13,14].

Currently, the most widely known and used surgical robot system is the Da Vinci Surgical System (Intuitive Surgical Inc., Sunnyvale, CA, USA) which is used in many branches of surgery, particularly urology and cardiac surgery. There are also other systems including CMR Surgical’s Versius, Medtronic’s Hugo, Zimmer Biomet’s ROSA, and Johnson & Johnson’s MONARCH, the latter being an example of integration with artificial intelligence. In this field of maxillofacial surgery, three approved robotic surgery systems are available: the Da Vinci Intuitive system, the Medrobotics Flex robotic system for TORS, and the Medineering robotic endoscopic guidance system [15,16,17,18,19,20].

Generally, surgical robots consist of a console, through which the surgeon controls the robot using a special joystick and foot pedals in order to manipulate surgical instruments, robotic arms, equipped with surgical instruments, a 3D vision system, with the aim of improving the surgeon’s visibility, and advanced software very often integrated with artificial intelligence algorithms to improve and optimize the movements of the robotic arms [21].

Robotic surgery is proving to be a useful technology for the surgeon particularly in performing complex surgeries where access is needed in small and delicate anatomical areas. For this reason, one of the most interesting regions for its application in maxillofacial surgery is the orbit. Owing to its anatomical peculiarities, namely a stiff osseous cone in which multiple, densely organized structures are present, the orbit represents a solid benchmark to implement the advantages offered by robotic surgery. This comprehensive review aims to analyze, through the study of the available literature, the possible applications of this technology in the treatment of surgical pathologies of the orbit, understanding its advantages and disadvantages, the most used robotic stations, and the clinical conditions for which robotic surgery is adopted.

## 2. Materials and Methods

Assuming a limited amount of evidence for this topic, the research query of this review was constructed by conducting a cross-sectional search on multiple databases to answer the following question: “What are the applications of robotic surgery in the management of orbital pathologies?”

### 2.1. Search Strategy for Identification of Studies

The query was composed using a mixture of Boolean operators and special characters as follows: (robot*) AND (orbital OR orbit) AND (surgery OR intervention OR surgical) NOT (space OR spatial). The latter operator excluded any item related to space research which could be mistakenly confused with the term “orbit”.

The MeSH database was used to improve the specificity of the query by combining the following search items: “Orbit” [Mesh] AND “surgery” [Subheading] AND “Robotics” [Mesh].

The literature search was conducted across the following databases: PubMed, CINAHL, Cochrane Central Register of Controlled Trials (CENTRAL), ClinicalTrials.gov, ScienceDirect, and Embase. Time constraints for the search included a frame between 30 April 2025 and 1 January 2015, thus including a time span of 10 years useful to capture the most recent innovations in robotic surgery.

The articles found in the databases consulted were saved in a unique BibTeX (.bib) file. This file was then imported into last version of Zotero (Corporation for Digital Scholarship, Vienna, VA, USA), where articles were organized and duplicates were eliminated.

### 2.2. Study Inclusion Criteria and Analysis

The types of included articles were original papers: clinical trials, randomized clinical trials, cohort studies. Given the limited amount of results expected, low evidence design studies such as small-sampled case series and case reports were also included. Search was limited to articles that evaluated the use of robotic surgery for pathologies affecting the orbital region in humans. Articles dealing with the use of robotic surgery in pathologies not involving the orbital region, articles without an abstract, reviews, systematic reviews, and meta-analyses were excluded. English was set as the only accepted language to select related articles.

The two researchers independently (Lo.M. and Lu.M) applied the inclusion and exclusion criteria above on the collected articles through an initial evaluation on the title and abstract and then through a detailed analysis of the full text of the articles, and in case of doubt, a third investigator (A.T.) was included in the evaluation. This review is limited to studies published in the last 10 years and in the English language.

**Figure 1:**
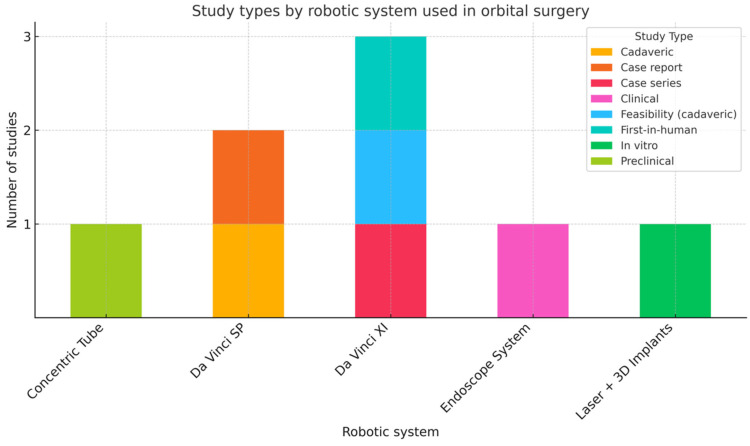
Graphical representation of the study types and robotic systems used across the included studies.

The results of the included studies are summarized in Table 1 and a graph designed to illustrate the robotic stations assessed in the literature (Figure 1) was created to compare the robotic systems used across the different type of studies. When multiple values were available for the same parameter, data were reported as mean (standard deviation (SD) or range) or median (interquartile range (IQR) or range).

**Figure 2:**
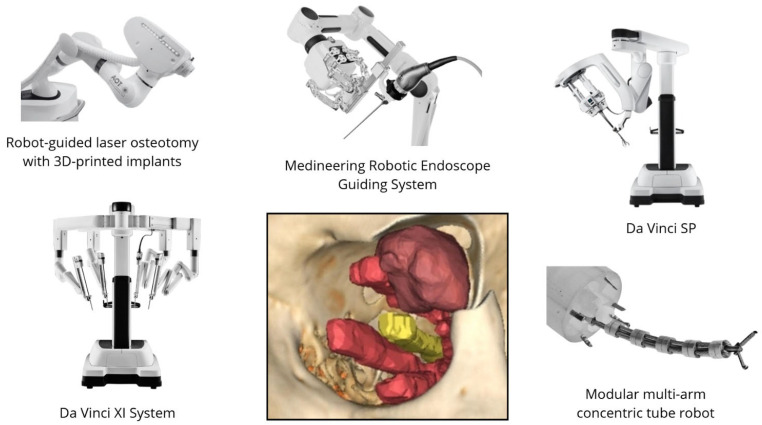
Robotic systems for orbital phatology reported across the included studies (image created with last version of Canva^®^).

Figure 2 provides a visual representation of the selected surgical robotic systems whose use in orbital pathology is reported in the literature.

## 3. Results

Results of the search process yielded a total of 491 studies which were initially retrieved by the investigators. After the identification of duplicates, 28 studies were removed. The remaining 463 studies were screened by title, and a further 434 studies were excluded. Subsequent screening by abstract led to the exclusion of an additional 15 reports. The full texts of 14 studies were analyzed, of which six did not fulfill the inclusion criteria and were removed. Finally, eight studies meeting the inclusion criteria were included in the review.

Studies considered by this comprehensive review explored various robotic systems and techniques applied in orbital surgery, encompassing cadaveric, in vitro, preclinical, and early clinical settings. The procedures investigated included fronto-orbital advancement, orbital and peri-orbital tumor resection, orbital decompression, and others such as lacrimal gland dissection and biopsy, medial and lateral orbital wall dissections, enucleation, and lid-sparing orbital exenteration.

### 3.1. Preclinical, In Vitro, and Cadaveric Studies

Maintz et al. (2025) [22] evaluated a system combining robot-guided laser osteotomy (CARLO^®^) and patient-specific 3D-printed PEEK (polyetheretherketone) implants for frontal-orbital advancement in an in vitro model. Using CT-based preoperative planning, the robot executed laser cuts with a median deviation of 0.44 mm from planned osteotomies. Implant fit was confirmed visually and digitally via image fusion. The system showed high reproducibility and accuracy, though limited access to the orbital cavity and laser beam line-of-sight restrictions were major challenges in replicating deep osteotomies [22].

Lee et al. (2024) [24] demonstrated the feasibility of robot-assisted lateral transorbital access to Meckel’s cave using the Da Vinci Xi in a cadaveric model. While adequate visualization and instrument movement were achieved after orbital rim removal, the approach was limited by narrow entry space and large instrument size. The study suggests future potential with smaller, specialized tools [24].

Faulkner et al. (2024) [25] assessed the Da Vinci SP (single-port) system in a cadaveric study involving a series of orbital approaches and techniques (lacrimal gland dissection and biopsy, medial and lateral orbital wall dissections, enucleation, and lid-sparing orbital exenteration). The SP system consists of a teleoperated robotic surgical device with a 28 mm diameter cannula which encases three multidirectional instruments and a 3D stereoscopic endoscope which provides depth to the surgeon’s console. Key findings included successful exposure of orbital fat, periorbita, and optic nerve regions; notably, one of the most appreciated features was third-arm soft tissue retraction. However, instrument articulation was restricted in the posterior orbit and the instrument diameter limited dexterity, particularly near the apex, where a bleeding emergency would be hardly managed in an endoscopic view [25].

Bruns et al. (2021) [29] developed a modular, multi-arm concentric tube robot system, designed to navigate the narrow, curved transnasal corridor to the orbital apex. The system integrated concentric pre-curved nitinol tubes and was capable of independent actuation at each arm. Navigation and targeting accuracy were evaluated using phantom models and cadaveric specimens. The robot achieved precise maneuvering within the orbit, demonstrating feasibility for future applications in tumor resection near the orbital apex. However, this study lacked in vivo validation, and no quantitative performance metrics (e.g., target registration error or execution time) were reported [29].

### 3.2. Clinical Case Reports and Series

Malik et al. (2024) [23] reported a clinical case series (n = 4) of robot-assisted resection of advanced periocular malignancies using the Da Vinci SP system. The procedures involved lower eyelid, medial canthus, and orbital rim resections with curative intent. The SP system enabled wide local excisions with preservation of the globe in all patients. Mean operative time was not reported, but no intraoperative conversions or serious complications occurred. One patient developed cervical lymph node metastasis requiring further treatment. Histologically clear margins were achieved in all four cases [23].

Jeannon et al. (2023) [26] described the use of the Da Vinci SP system for robot-assisted orbital oncology surgery (RAOS) in a single-patient case. The approach allowed en bloc tumor resection with preservation of visual acuity and extraocular movements. The system’s articulated arms allowed precise dissection around the tumor with minimal collateral tissue manipulation. The patient experienced no perioperative complications. However, as a single-case report, the clinical reproducibility and generalizability of this technique remain uncertain [26].

Wang et al. (2022) [27] documented the first in-human application of robot-assisted orbital fat decompression surgery using the Da Vinci Xi system. The approach was transconjunctival, and robot-assisted instruments were used to resect intraconal and extraconal fat in patients with thyroid eye disease. All patients had significant reduction in proptosis (mean ~3 mm) and no major complications. Robotic access improved visualization and ergonomics for deep orbital fat removal. However, the sample size was small, and follow-up was limited to early postoperative results [27]. Future studies are warranted to investigate whether the use of pharmacological treatments, such as teprotumumab [30], in conjunction with surgery could further facilitate and improve surgical outcomes.

Mattheis et al. (2021) [28] reported the first use of the Medineering Robotic Endoscope Guiding System, which assists in endoscopic orbital decompression. The system allowed precise positioning and stabilization of the endoscope, freeing the surgeon’s hands for bimanual dissection. The setup was compatible with existing endonasal techniques and allowed two- and four-handed workflows. The authors noted increased overall operative time due to setup and calibration, but the exact duration was not reported [28].

## 4. Discussion

Orbital robotic surgery represents an advanced frontier in surgical operations involving the orbital region, characterized by narrow spaces, delicate anatomical structures and the need for absolute precision. Thanks to technological advances, robot-assisted systems, such as the Da Vinci Xi, are increasingly being used to treat orbital pathologies, particularly in oncological and functional settings. These systems offer high-definition three-dimensional vision, precision in movement, and scalability of movements that overcomes the limitations of traditional manual instruments.

### 4.1. Indications for Orbital Robotic Surgery

The indications for the use of orbital robotic surgery are mainly focused on orbital tumors, orbital fat decompression, and other pathologies requiring precise intervention in confined spaces. Tumors that develop in the orbital region, such as advanced periocular tumors, can benefit from the use of robotics for gentle resection without compromising the globe or the optic nerve [23]. In addition, orbital decompression, a procedure frequently performed in patients with Graves’ ophthalmopathy, can benefit from robotic assistance to improve visibility and precision while minimizing tissue damage [27].

### 4.2. Robotic Surgical Approaches

Several approaches have been explored in robot-assisted orbital surgery. Among the most relevant are the transnasal and transorbital approaches, each with its own specific applications. The transnasal approach, which allows access to the orbital apex, is particularly useful for resection of posterior orbital tumors due to the ability, in particular in the case of concentric tube robots, to navigate around anatomical obstacles and reduce the risk of accidental injury [29]. The lateral transorbital approach, although promising, is still in its development phase, as currently available robotic instruments are relatively bulky, limiting access to narrower spaces. However, future developments in terms of instrument miniaturization could significantly improve this approach [24]. Overall, these approaches offer good functional and aesthetic results [28].

### 4.3. Advantages of Orbital Robotic Surgery

Orbital robotic surgery offers numerous advantages over traditional techniques. Among the main ones are improved precision, reduced tremor, and a three-dimensional view that allows a more accurate assessment of the orbital region [23,26]. The ability to scale the surgeon’s movements allows gentle manipulation of tissue without damaging vital structures such as the optic nerve, extraocular muscles, or blood vessels [27]. In addition, improved ergonomics for the surgeon, thanks to the remote console, reduces the risk of fatigue and improves operational precision [27].

Furthermore, robotic surgery minimizes incision and tissue manipulation, reducing the risk of post-operative complications and improving recovery time. In patients undergoing resection of orbital tumors or orbital fat decompression, this approach has led to faster recovery, with a significant reduction in post-operative pain [26].

### 4.4. Disadvantages and Limitations of Orbital Robotic Surgery

Despite its many advantages, orbital robotic surgery still has some limitations (Table 2). One of the main ones is the size of the instruments, which can be too large for spaces as narrow as the orbit. Currently, robotic systems such as the Da Vinci Xi and other instruments have diameters that do not easily fit into the limited spaces typical of the orbital cavity [23,24]. In addition, the lack of tactile feedback, coupled with the need for an assistant to ensure protection of the globe and soft tissue during surgery, is another significant disadvantage [23,27]. Despite this, trials have already been initiated to overcome these limitations such as the implementation of haptic feedback and the reduction in instrument size [29].

Another important consideration is that, although robotic systems enhance surgical visibility and precision, they require a high level of surgical expertise and involve a steep learning curve. Effective use also demands familiarity with specific instruments and often extensive preoperative planning [28]. Furthermore, the preparation time for robotic procedures is typically longer compared to conventional surgery, despite the intraoperative benefits they offer [27]. Finally, the high cost of robotic platforms remains a significant barrier, limiting their widespread institutional adoption. To address this, it would be beneficial to promote the shared use of robotic systems across different departments within hospitals, optimizing resource allocation and improving cost-effectiveness.

### 4.5. Future Perspectives and Developments

Despite its current limitations, robotic surgery—particularly in the orbital region—is continuously evolving. Technological innovations such as the miniaturization of robotic arms, flexible instrumentation, and the integration of artificial intelligence (AI) are paving the way for safer and more precise procedures [24,28]. These advances are particularly relevant in orbital surgery, where narrow anatomical spaces require delicate manipulation.

Moreover, ongoing preclinical and early-phase clinical trials are exploring novel approaches and robotic platforms (e.g., concentric tube robots and the Da Vinci SP system) that aim to overcome spatial constraints and improve access to posterior orbital regions [23,27,29]. For instance, the concentric tube robot has demonstrated promising results in cadaveric models for accessing the orbital apex via transnasal routes [29].

However, in parallel with technical progress, there are regulatory and logistical hurdles that may delay widespread adoption. These include regulatory approval pathways for new robotic platforms, standardization of surgical protocols, and validation of clinical efficacy through large-scale trials. Additionally, the high capital investment and ongoing maintenance costs pose a challenge. Addressing these issues will require coordinated efforts between device manufacturers, clinical researchers, and regulatory bodies.

With continued innovation and multidisciplinary collaboration, robotic surgery is expected to become an increasingly viable and beneficial option for orbital pathologies, offering improved precision, reduced morbidity, and enhanced surgical outcomes.

### 4.6. Applications of AI in Robotic Surgery

The integration of artificial intelligence into robotic surgery has brought important benefits. Regarding preoperative planning AI algorithms can analyze preoperative imaging and patient data to create detailed surgical plans. These plans can include optimal incision points, predicted tissue responses, and potential risk factors. By simulating the surgery beforehand, surgeons can anticipate challenges and refine their approach, leading to better outcomes. A further application of this technology is intraoperative care. In fact, during surgery, AI systems provide real-time assistance by analyzing live data from the surgical field. For example, AI can monitor vital signs, detect changes in tissue morphology, and alert surgeons to potential complications such as bleeding or organ perforation. This real-time feedback enhances the surgeon’s ability to make informed decisions quickly.

Finally, the successful integration of AI into surgical practice hinges on surgeon training and adaptation. As these technologies evolve, surgeons must acquire new skills and learn to collaborate effectively with AI systems, all while maintaining their critical role as the primary decision-makers in the operating room. Addressing these challenges holistically is essential to unlocking the full potential of AI in robotic surgery while safeguarding patient trust and outcomes.

## 5. Conclusions

This comprehensive review highlights the growing application of robot-assisted techniques in orbital surgery, particularly in complex anatomical areas that demand high precision. The included studies demonstrate that robotic systems, such as the Da Vinci platforms and concentric tube robots, offer notable advantages in terms of surgical accuracy, visualization, and tissue preservation. These benefits translate into improved functional and aesthetic outcomes and potentially faster recovery times. However, current limitations, including the size of instruments, lack of tactile feedback, and extended setup times, pose challenges to their widespread adoption. Moreover, most available evidence remains limited to early-phase, cadaveric, or small-scale clinical studies. Further advancements in miniaturization, system integration, and clinical validation through larger trials will be essential to fully establish the role of robotic surgery as a standard in the treatment of orbital pathologies.

## Figures and Tables

**Table 1:** Selected studies. VSP: virtual surgical planning; RMS: root mean square; IQR: interquartile range; SCC: squamous cell carcinoma; BCC: basal cell carcinoma; SP: single-port.

Study	Robot/System Used	Study Type	Target Pathology/Procedure	Key Findings	**Limitations**
Maintz et al. (2025) [22]	Robot-guided laser osteotomy with 3D-printed implants.	In vitro accuracy assessment.	Fronto-orbital advancement.	Achieved high accuracy in bone cutting and positioning; osteotomy deviations from VSP indicated max distance of 1.7 mm, median deviation of 0.44 mm, and a max RMS error of 0.67 mm.	Accessing the orbital cavity with the laser remained challenging.
Malik et al. (2024) [23]	Da Vinci Xi system.	Clinical case series, four patients, mean age 61 (IQR, 31) years.	Resection of advanced periocular tumors (two SCCs, BCC).	Successful globe-sparing resections; preservation of vision; one patient required additional procedures for lymph node involvement.	Small sample size; further studies needed to confirm findings.
Lee et al. (2024) [24]	Da Vinci Xi system.	Feasibility, cadaveric.	Lateral transorbital approach to Meckel’s cave.	Robotic access feasible with one tool + camera; good visualization and working angles after orbital rim removal.	Limited entry space; only one tool insertable; tool size too large; lacks haptic feedback.
Faulkner et al. (2024) [25]	Da Vinci SP.	Cadaveric study.	Soft tissue orbital procedures (lacrimal gland dissection and biopsy, medial and lateral orbital wall dissections, enucleation, and lid-sparing orbital exenteration).	All seven procedures completed successfully without complications; setup and operative times were acceptable.	Instrument size limited surgical access and precision, particularly at the orbital apex.
Jeannon et al. (2023) [26]	Da Vinci SP.	Clinical case report.	Orbital oncology surgery (medial canthal BCC recurrence).	En-bloc resection of tumor with preservation of eye movements and vision; uneventful recovery.	Single case; broader applicability requires further study.
Wang et al. (2022) [27]	Da Vinci Xi system.	First-in-human study.	Orbital fat decompression surgery.	All procedures successfully performed; significant reduction in exophthalmos; no complications reported.	Limited to initial human trials; larger studies required for validation.
Mattheis et al. (2021) [28]	Medineering Robotic Endoscope Guiding System.	Clinical study, eight patients.	Endoscopic orbital decompression.	Found to be a safe and effective support in endoscopic skull base surgery, allowing two-handed or even four-handed settings.	Increased duration of surgery due to setup time.
Bruns et al. (2021) [29]	Modular multi-arm concentric tube robot.	Preclinical (cadaveric).	Transnasal access to orbital apex tumors.	Demonstrated feasibility of accessing the orbital apex with a concentric tube robot system.	Not tested in live human subjects; clinical efficacy not established.

**Table 2:** Advantages and disadvantages of orbital robotic surgery.

Advantages	Disadvantages
Improved precision Reduced tremor Three-dimensional view Gentle tissue handling Less damage to delicate tissues such as optic nerve, muscles, and vessels Improved ergonomics Smaller surgical incision Fewer postoperative complications Less surgical time Faster post-surgical recovery Reduction in postoperative pain	Size of instruments Lack of tactile feedback Need for assistant High level of competence and learning curve Increased time for preparation for surgery Robotic systems cost

## Data Availability

Not applicable.
